# Soft Robotics: A Systematic Review and Bibliometric Analysis

**DOI:** 10.3390/mi14020359

**Published:** 2023-01-31

**Authors:** Dan-Mihai Rusu, Silviu-Dan Mândru, Cristina-Maria Biriș, Olivia-Laura Petrașcu, Fineas Morariu, Alexandru Ianosi-Andreeva-Dimitrova

**Affiliations:** 1 Mechatronics and Machine Dynamics Department, Technical University of Cluj-Napoca, 400114 Cluj-Napoca, Romania; 2 Department of Industrial Machines and Equipment, Engineering Faculty, Lucian Blaga University of Sibiu, Victoriei 10, 550024 Sibiu, Romania

**Keywords:** soft robotics, bibliometric analysis, actuators, manufacturing technology, material, sensor, modeling methods

## Abstract

In recent years, soft robotics has developed considerably, especially since the year 2018 when it became a hot field among current research topics. The attention that this field receives from researchers and the public is marked by the substantial increase in both the quantity and the quality of scientific publications. In this review, in order to create a relevant and comprehensive picture of this field both quantitatively and qualitatively, the paper approaches two directions. The first direction is centered on a bibliometric analysis focused on the period 2008–2022 with the exact expression that best characterizes this field, which is “Soft Robotics”, and the data were taken from a series of multidisciplinary databases and a specialized journal. The second direction focuses on the analysis of bibliographic references that were rigorously selected following a clear methodology based on a series of inclusion and exclusion criteria. After the selection of bibliographic sources, 111 papers were part of the final analysis, which have been analyzed in detail considering three different perspectives: one related to the design principle (biologically inspired soft robotics), one related to functionality (closed/open-loop control), and one from a biomedical applications perspective.

## 1. Introduction

The field of soft robotics is scientifically considered a field of spectacular development from one year to the next, this being based on the potential that it has, namely, to offer other perspectives in the field of robotics and many others. What is spectacular is that the field of soft robotics, being relatively young and appearing as a term only in 2008, has gradually developed, reaching over 1000 scientific publications in databases such as Web of Science (WOS) and Scopus in the year 2022. Several aspects related to the history of soft robots were addressed in the review by Bao et al. [1]. Since the field of soft robotics is young, open, and outside of dogmatic restrictions in terms of manufacturing, modeling, and fields of use [2], this can introduce several ambiguities or confusions. One of these is related to the definition of soft robotics. In the specialized literature analyzed, many authors propose their definitions based on their research, but the soft robotics community has not reached a unanimously accepted definition that answers the question concerning what soft robotics is. That is why in this paper some of the definitions are accumulated, giving young or senior researchers a perspective on the mentioned question. The first such definition is: “*Soft robots are primarily composed of easily deformable matter such as fluids, gels, and elastomers that match the elastic and rheological properties of biological tissue and organs.”* [2]. The following definition is provided by Rus et al.: “*We define soft robots as systems capable of autonomous behavior and which are primarily composed of materials with modules in the field of soft biological materials.”* [3]. Alternatively, the definition from Panagiotis Polygerinos et al. states that a “*soft robot is appropriately named when the stresses it is subject to cause it to deform prior to damaging the class of objects for which it was designed (whether it be human or cantaloupe); we acknowledge that traditional robots can be thought of as soft when interacting with a harder object, such as a diamond.*” [4]. At the same time, the following definition was also offered: “*Soft robotics is the subject to study how to make use of the softness of an object or a piece of materials or a system for building a robot by satisfying a required softness to both its environment and its receiver.”* [5]. There is also the definition from Liyu Wang et al.: “*We define soft-matter robotics as robotics that studies how deformation of soft matter can be exploited or controlled to achieve robotic functions.”* [6]. These definitions of soft robotics contain similar aspects related to the source of inspiration, material, high compliance, and high deformability of soft robots. Considering the definitions above, one can be proposed that integrates all the aspects identified. A possible collective definition could be the following: soft robotics is a growing subfield of robotics that mainly draws inspiration from biological systems and uses materials with coefficients in the range of soft materials with high and continuous deformability so as to achieve specific robotic functions.

Over the years, in the soft robotics literature, several reviews have been published that address the field and focus on different specific application areas or reviews so as to create a comprehensive and precise picture. Based on the accelerated growth of scientific publications in recent years, the present paper responds to the need for centralization and provides an updated perspective of the achievements of recent years by generating a comprehensive view of the field. This paper represents a hybridization that approaches two categories of analysis. In the first part of the paper, a bibliometric analysis is carried out in which the evolution of the number of scientific publications from 2008 to July 2022 is identified alongside an analysis of the publications that considers aspects such as the most productive articles, journals, countries, and authors in this field, as well as the most cited scientific articles. The second part of the paper analyses the state of the art in the field of soft robotics from 2018 to July 2022, whereby the selection of articles is based on a clear methodology that is carried out in two stages due to the large number of articles found. 

Considering the first part of the research, other reviews with bibliometric or scientometric analyses of soft robotics have been identified in the literature. This tool provides authors with a relevant method for mapping the evolution of the number of scientific publications over time in various fields. The first identified bibliometric analysis conducted in the field of soft robotics was that of Bao et al. [1], who retrieved data from the WOS database for studies published between 1990 and May 2017 using a range of keywords relevant to the field, which resulted in 1495 review and research articles being selected; in that paper numerous different aspects were analyzed, such as those related to productive countries, collaborations between countries, universities, journals, productive authors, and research areas contributing to the field. Another review that treats the field of soft robotics from a quantitative perspective is that of Yitong Zhou et al. [7], who conducted a scientometric analysis of studies published between 2010 and July 2021 (also from the WOS database) using a series of domain-specific keywords. From the search, 10504 results were obtained, and the researchers analyzed similar aspects to those in the analysis of Bao et al. In that paper, CiteSpace was used to make co-citation network maps. Another graph that highlights the evolution of the number of scientific publications is that of Laschi et al. [8], whose study was based on the Scopus database and publications between 2004 and 2016.

The second part of the paper, which qualitatively analyses the field of soft robotics, represents the state of the art in the field. The analysis of the field is based on 6400 research and review articles selected from four databases (WOS, ScienceDirect, IEEEXplore, and SpringerLink) with multidisciplinary character and the journal “*Soft Robotics*”. All these articles were obtained with an exact match for the search term “Soft Robotics” in the 2018–July 2022 timeframe. Due to the large number of results identified, the selection methodology was based on a set of clear inclusion and exclusion criteria, with the selection of relevant articles being carried out in two stages. After the first selection stage, 824 articles were selected based on the exclusion criteria. Following the second selection stage, 111 relevant articles were selected by applying the inclusion criteria that needed to be satisfied for articles to be part of the final domain analysis.

## 2. Bibliometric Analysis of the Field of Soft Robotics

### 2.1. Selection Methodology

The bibliometric analysis considering the evolution of the number of publications is based on publications related to soft robotics between 2008 and July 2022. The year 2008 was not chosen by chance, as this was the year when the term soft robotics was widely adopted by the robotics community. For the graph regarding the mentioned evolution there were four databases used (WOS, ScienceDirect, IEEEXplore, and SpringerLink), as well as the specialized journal “*Soft Robotics*”. The data from the mentioned sources were retrieved with the exact search term “Soft Robotics”, which best characterizes the domain. Only reviews and research articles in English were selected. For the bibliometric analysis considering aspects such as authors, countries, and journals, the data were retrieved from the WOS Core Collection database with the same inclusion criteria as above.

### 2.2. Results

**Figure 1:**
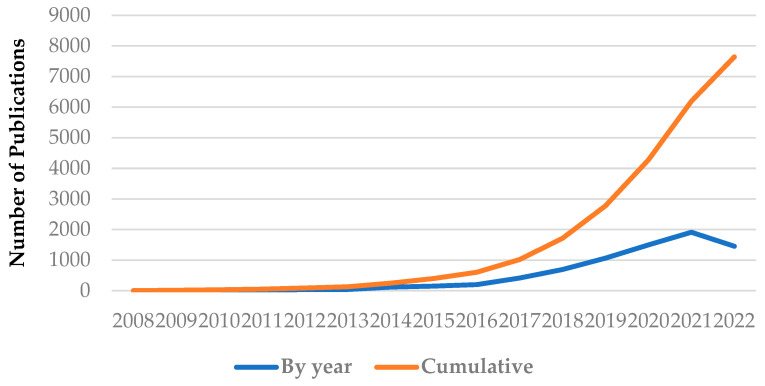
Evolution of scientific publications in the 2008–July 2022 period with the exact search “Soft Robotics” on the Science Direct, WOS, IEEE Xplore, and SpringerLink databases and the “*Soft Robotics”* journal.

The first analysis carried out within the bibliometric study is related to the evolution of the number of publications (Figure 1) in the field of soft robotics from the mentioned databases and the journal “*Soft Robotics*”. As a result of the analysis, 7646 publications were obtained. To avoid journals found in multiple databases, 35 journals that were duplicates were excluded from the analysis of the WOS database. This approach is an original one because, compared to other scientific sources, there is no such analysis in which the data are taken from several databases.

The graph shows two curves that represent the annual evolution, which represents the results for each year from the four databases and the journal (blue line), and the cumulative evolution, which represents the summation of all the articles found each year from the four databases and the journal (orange line). The field of soft robotics started timidly with only a few articles in 2008 and continued with a weak evolution until 2012–2013 when the number of publications began to grow at a higher rate, though far from reaching 1000 articles. The increases in 2012–2015 are somewhat constant and from 2016 the domain begins to have a strong increase in the number of articles; in 2017 the domain accumulated more than 1000 articles. From 2016 to 2021, the number of articles grew significantly from year to year, which shows the interest of more and more researchers in this field. In 2021, the number of published articles reached approximately 2000, and this trend continued in 2022 with approximately 1500 articles being recorded by July 2022. What can be observed from the graph in Figure 1 is that an incredibly large number of publications were published in the 2018–2022 period. Publications from 2008–2017 represent 13.37% of the production of articles in the field, while those from 2018–2022 represent 87.63% of the entire 2008–2022 period.

The second analysis in the bibliometric study was conducted based on the WOS database, which is an international multidisciplinary database that gives the field of soft robots a global presence. It also provides researchers with a range of criteria for analysis according to their field of interest, ranking search results according to criteria selected by the user. After applying the criteria mentioned in the selection methodology section, a total of 3681 research articles and reviews were obtained from the WOS Core Collection database. Analyzing the 3681 articles according to the two types of documents selected as filters, research articles predominate with 3338 articles, representing 90.67%, and 343 review articles represent 9.32% of the total. This distribution of the number of articles represents a typical one, with review articles usually having a smaller number of publications. However, the field of soft robotics is continuously evolving. In a very short time window, as illustrated by Figure 1, many new developments were documented by new research articles; as a consequence, many past reviews of the field have lost their edge. The ones that are still relevant approached the subject with a different methodology. Thus, the aim of this review article is to provide a fresh and valuable perspective.

As soft robotics is a multidisciplinary field [3], in recent years this feature has been further extended. Table 1 shows the top 10 WOS research areas ranked by the number of articles. The main category is “Materials Science Multidisciplinary”, which consists of 1335 publications representing 36.267% of the 3681 articles. A considerable amount of soft robotics features is based on material properties such as compliance, elasticity, and high and continuous deformability. The second significant research area is “Robotics”, with 1080 articles representing 29.340% of the 3681 results. A total of 650 papers that contributed to the field of soft robotics were from the “Nanoscience Nanotechnology” category. The research contribution indexed in the “Nanoscience Nanotechnology” category in the field of soft robotics addresses aspects related to materials, actuators, and sensors. The multidisciplinary nature of soft robotics also includes areas such as “Applied Physics”, “Chemistry”, and “Electrical Engineering”. 

Considering the most productive journals publishing on soft robotics, Table 2 shows the top 10 journals in this area. The journal “*Soft Robotics*” ranks first with the highest number of articles published, namely 457. This journal is dedicated to this field and has published six issues of the journal every year since 2018. This journal accounts for 12.415% of the identified articles, which is a significant percentage. The “*ACS Applied Materials and Interfaces*” journal is the second-ranked journal with 179 publications (4.863%), which indicates a significant difference between the top two places. As “*ACS Applied Materials and Interfaces*” is not a soft robotics journal, it publishes specialized material articles. “*IEEE Robotics and Automation Letters*” was ranked in 3rd place and is a journal that is focused on robotics and automation, though it also publishes articles related to soft robotics. In the 4th place, the “*Advanced Materials*” journal focuses on materials and therefore publishes articles in the field of soft robotics from a materials perspective. Each journal has more than 100 articles published on soft robotics, representing more than 3% of the 3681 articles.

Looking at the other positions, there is an alternation between material-focused journals and smart systems, robots, and AI. Referring to the impact factor of each journal, “*Advanced Materials*” has the highest impact factor (32.09) and “*Advanced Functional Materials*” also has a high impact factor (19.92), both journals being focused especially on materials. The robotics journal with the highest impact factor is “*Soft Robotics*” (IF 7.784), while it also has the highest contribution to the field in terms of the number of articles.

Table 3 identifies the 10 countries that made the most substantial contribution to soft robotics. More than 60% of articles come from authors belonging to the People’s Republic of China (1183 items representing 32.138%) and the USA (a percentage close to that of China with 1160 items representing 31.513% of the total). A likely reason attributed to the productivity of these countries is that these countries have several strong funding programs dedicated to soft robotics that are supported by their governments, such as DARPA ChemBots in the US or Tri-Co Robot in China; however, the main reason resides in the fact that both the USA and China have a large demographic involved in research, which allows them to publish a large number of papers in all fields, especially in new and emerging ones. The rest of the top countries each contribute less than 8%, and these countries are largely in either Europe or Asia. European countries such as England, Italy, Germany, and Switzerland account for 23.554% of articles, i.e., 867 articles, and Asia contributed 49.306% of articles, i.e., 1815 items. 

Analyzing the results according to the most productive authors in the field, Table 4 shows the top 10 authors with the highest number of articles. Majidi (USA) is the most productive author with 39 papers representing 1.059% of the total. Close behind in 2nd, 3rd, and 4th place are the Italian authors Cianchetti, Laschi, and Mazzolai with 38, 38, and 35 articles, respectively. In 5th and 6th place are two authors from China with 34 and 32 articles, followed in 7th and 8th place by two authors from the USA with 31 and 29 articles.

Table 5 identifies the most cited articles in the WOS database for the 2008–2022 period. Table 5 also identifies the journal in which the article was published, the year of publication, the author, the country, the title of the article, and, of course, the number of citations in WOS. The most cited article in WOS is by Rus et al., with a citation count of 2596. This article was published in 2015 in the journal “*Nature*” with the title “Design, fabrication, and control of soft robots”; this is a review article providing an overview of the field of soft robotics [3]. Since its publication, this article has had a strong impact on the scientific community in the field, recording the highest increase in citations reported in a year [1]. In second place with 1641 citations is the review by Amjadi et al. titled “Stretchable, Skin-Mountable, and Wearable Strain Sensors and Their Potential Applications: A Review” [9], which was published in 2016 in “*Advanced Functional Materials”*. Another review article is ranked third with 1268 citations and was written by Shepherd et al. The article is titled “Multigait soft robot” and was published in “*Proceedings of the National Academy of Sciences of the United States of America”* in 2011 [10].

The 4th, 5th, and 6th place articles are occupied by three US authors who have over 1000 citations each, namely 1109, 1096, and 1033. These articles were published in the years 2013, 2011, and 2018. The 4th ranked article is a review and is titled “Soft robotics: a bioinspired evolution in robotics” [11], which was published in the journal “*Trends in Biotechnology*”. In fifth place is the article published in the journal “*Angewandte Chemie-International Edition*” titled “Soft Robotics for Chemists.” [12], and in sixth place is the article “Skin electronics from the scalable fabrication of an intrinsically stretchable transistor array” [13], which was published in the journal “*Nature*”. Tee et al. is another group of Singaporean authors with over 1000 citations, more precisely 1032. Their article was published in 2012 in the journal “*Nature Nanotechnology*” and occupies 7th position; the article is titled “An electrically and mechanically self-healing composite with pressure- and flexion-sensitive properties for electronic skin applications” [14]. The remaining positions (8, 9, and 10) are occupied by three authors from the USA who have less than a thousand citations, namely 792, 790, and 767. Their articles were published in journals dedicated to materials and one of them was published in the journal “*Nature*”. The three articles are “Stretchable and Soft Electronics using Liquid Metals” [15], “Printing ferromagnetic domains for untethered fast-transforming soft materials” [16], and “Pneumatic Networks for Soft Robotics that Actuate Rapidly” [17].

## 3. State of the Art in Soft Robotics

This chapter is part of the second section of this work that represents the qualitative component, which attempts to create a global but comprehensive picture of the field of soft robotics. As mentioned in chapter 2 of the bibliometric analysis of this paper, the accelerated growth and large number of articles found in the literature in the field achieves this rather challenging goal. Given the current context, a clear and objective methodology for the selection of bibliographical references is required to identify and select relevant bibliographical references. In addition to the attention paid to the methodology of reference selection, analysis of the selected bibliographic references was paid due attention to as well, with each part of the paper being analyzed in detail so that a variety of characteristics specific to soft robots could be documented in tabular form.

### 3.1. Methodology for the Selection of Bibliographical References

In our approach to the selection of bibliographic references, four international databases and one journal in the field were chosen. The four databases were chosen with the intention of providing greater diversity within identified fields and applications, which was achieved by choosing databases with a multidisciplinary character (WOS and ScienceDirect) and databases that offer strong technical features (IEEEXplore and SpringerLink). The “*Soft Robotics*” journal was chosen since it only publishes articles in the field of soft robotics. All these databases were selected to increase the relevance of the study as well as to satisfy its multidisciplinary character.

This study was based on research articles and reviews written in English during the 2018–July 2022 timeframe. This range, according to the bibliometric analysis above, represents 87.63% of all research and review articles identified from the four databases and the journal. This confirms that the relevance of this study is significant. The exact search term chosen to identify relevant bibliographic references was “Soft Robotics”. This expression best characterizes the domain of the same name. In the database search field, the exact phrase was entered using quotation marks, and all results were sorted by their relevance while applying the criteria mentioned below.

**Figure 2:**
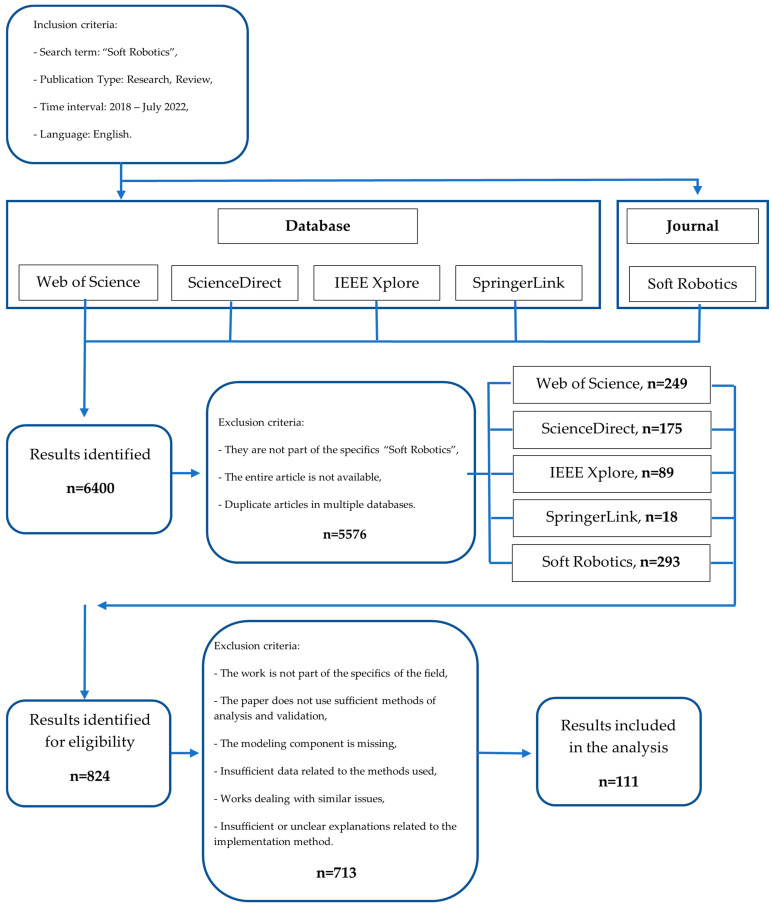
Graph of the selection process of the bibliographic references relevant to this analysis according to [18].

The search identified an impressive number of research articles and reviews, with 6400 results identified across the four databases and the “*Soft Robotics*” journal. Due to a large number of papers found, it was decided that the selection of articles would be carried out in two stages based on clear criteria. A graph of the search process is shown in Figure 2 (inclusion criteria). For the first selection stage, the eligibility criteria on which the selection of articles was based were related to the following:

- Specific characteristics of soft robots are identified;
- Materials and actuators are used that provide compliance to soft robots;
- Manufacturing methods, sensors, and domain-specific modeling methods are identified;
- The article clearly and concisely presents data on the structure of the article.

A total of 5576 articles were excluded in the first selection phase by following the eligibility criteria mentioned above. Analysis of the articles for selection was mainly based on a careful analysis of the abstracts of the articles and, to further increase the relevance of the study, a visual scan of the entire article was also performed. A significant number of duplicate articles were excluded from the analysis as they were found in several databases. Firstly, duplicate articles found in multiple databases were removed. Secondly, some articles were removed because the full article was not available, and most articles were removed because they did not deal specifically with the field of soft robotics. After the first selection stage, a total of 824 articles were obtained, which were analyzed in the second selection stage. 

A large number of publications was taken from the “*Soft Robotics*” journal. Additionally, a considerable number of publications were retrieved from the WOS and ScienceDirect databases, as these being databases contain an impressive number of publications.

In the second selection stage, 111 articles were selected from the 824 publications for state-of-the-art analysis. In this stage, the selection of articles was conducted according to detailed analysis of the whole article, and the selection was based on the following exclusion criteria:

- The work reviewed should clearly and sufficiently present the issues addressed;
- Diversity in soft robot applications;
- The variety of aspects related to materials, actuators, manufacturing technologies, sensors, and control systems used in the current soft robot framework;
- Aspects related to the mode and source of energy used in the operation of soft robots;
- Validation of the performance of soft robots through various numerical, experimental, or analytical analysis methods.

At this stage, 713 articles were excluded, with the majority of articles being excluded due to the following issues:

- Works dealing with similar issues;
- Insufficient or unclear explanations related to the implementation method;
- Insufficient data related to the methods used;
- The paper does not use sufficient methods of analysis and validation;
- The work is not part of the specifics of the field.

### 3.2. Analysis of Bibliographical References

Analysis of the bibliographical references was performed from the perspective of three different directions. We thus proposed the analysis of the selected publications from a perspective related to the design principles of soft robots (biologically inspired soft robotics), from the perspective of functionality (closed- or open-loop control), and from the perspective of applications (applications of soft robots in the biomedical field). With this approach we tried to capture new and valuable aspects compared to other review articles. We also approached the analysis of bibliographic references according to the components of soft robots that are presented in the tables in the appendix of the paper (Table A2, Analysis of bibliographic references according to the materials; Table A3, Analysis of bibliographic references according to the actuators; Table A4, Analysis of references according the specific technologies; and Table A5, Analysis of references according to the modelling methods; Table A6, Analysis of bibliographic references according to the sensors).

#### 3.2.1. Bio-Inspired Soft Robots

Biological organisms such as animals rely on the deformation of their body structure during locomotion. Their implicitly compliant deformable structure gives them efficient locomotion in the natural environments in which they live. These characteristics of living things have inspired engineers and researchers to integrate nature-inspired elements into their robotic structures, equipping robots with the ability to interact adaptively to unpredictable and unknown environments. Coyle et al. presented biologically inspired soft robots from a mechanical perspective, specifically related to design, material choice, and actuation [19]. Ren et al. compared the capabilities of soft robots to those of biological systems. According to them, there is still a large discrepancy between the two in terms of autonomy and integrated structures such that biologically inspired soft robots can only achieve “natural life artificially”. Some of these gaps are related to materials, control, and data processing algorithms, with flexible sensors and finite element simulation methods just some of the components of soft robots where significant developments are needed to realize bio-integrated and autonomous soft robots [20]. Mahdi et al. discussed publications from 2017 to 2020 from the perspective of the materials used in the realization of soft actuators and sensors. As for soft actuators, they have developed in terms of actuation parts and mechanical properties being improved; however, they are still yet to be integrated into industrial or commercial applications and improvements are still needed in terms of output force and limited lifetime. Regarding soft sensors, their accuracy, sensing range, and sensor linearity issues, they require additional analysis and modeling [21]. 

**Figure 3:**
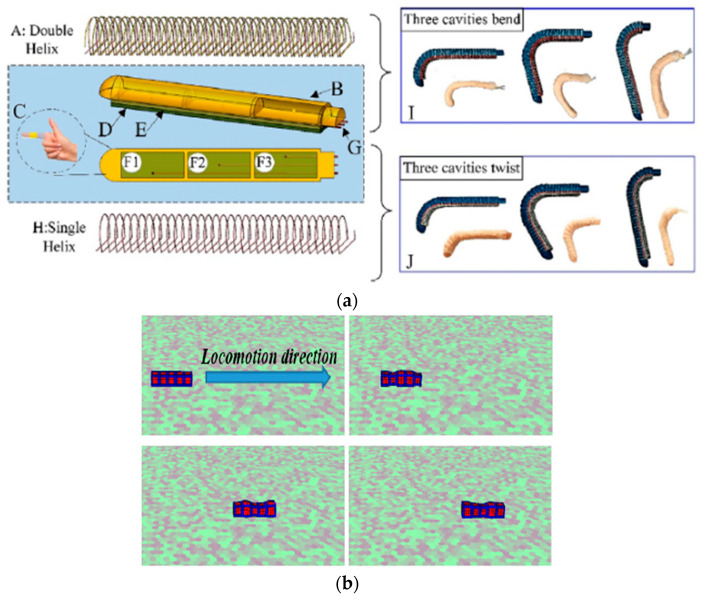
(**a**) Finger actuator structure; reproduced with permission from [28]; published by ELSEVIER, 2019; (**b**) modular robot simulated in VoxCAD software [30].

Liu et al. proposed a miniaturized bio-inspired robot with grasping capabilities and crawling and jumping locomotion capabilities in wet environments that can be used in medical applications such as drug delivery. The robot is based on a structure that has five layers, with each layer being 20 μm thick and possessing different functionalities when assembled. These layers include the pneumatically actuated actuator, as well as a layer with sensing properties that provides the possibility of closed-loop control [22]. Qin et al. also developed a crawling locomotion robot based on the use of springs and electrostatic actuators for legs that was vacuum-driven with fast locomotion and movement on vertical surfaces [23]. Guo et al. developed a soft robot with crawling realized through locomotion based on two EA legs, and the robot also had a dielectric elastomeric actuator inside that was a pre-tensioned spring that could help the robot during locomotion [24]. Another type was a bio-inspired robot with crawling locomotion that was driven by magnetic fields and which had PrFeB microparticles in the structure; this type of robot was made by V. K. Venkiteswaran et al. [25]. Niu et al. proposed a magnetically actuated crawling through locomotion robot that is not connected to an external component. The robot is driven by a rotating platform with permanent magnets that move constantly, namely by driving the robot in the direction of platform movement [26]. Zhang et al. proposed a soft robot inspired by the propulsion system of cuttlefish (cephalopods). It is based on a biomimetic siphon equipped with a diameter-varying pressure control channel, which represents the propulsion system, and the corresponding omnidirectional motion of orientation is achieved using three siphons positioned on the circumference of the propulsion siphon [27]. The issue of improving the lives of people with disabilities was addressed by Feng et al., who developed an artificial hand based on fluid actuators reinforced with fiber that contained three independently actuated cavities. This artificial hand was controlled by pressurization as well as by the capture of myoelectric hand signals by surface electrodes. The artificial hand’s control system is based on two control components, one corresponding to finger actuation by solenoid valves and pressure sensors and one corresponding to the human–computer interface seen in Figure 3 (a) [28]. Caterpillar locomotion was a source of inspiration for Zou et al., who developed a reconfigurable modular soft robot with omnidirectional locomotion composed of nine independent pneumatically actuated modules that was controlled via solenoid valves and pressure sensors that set the robot in motion according to the desired configuration [29]. Sui et al. simulated the behavior of a modular robot in VoxCAD software to validate the model and reduce design time, as shown in Figure 3 (b) [30]. Caterpillar locomotion also inspired Li et al., who developed a soft unconnected robot with a dielectric elastomer-based drive that moves at a speed of 100 mm/s [31]. Li et al. also developed a series of robots with actuators based on dielectric elastomers that can move at a speed of 0.65 m/s with a diameter of 106 mm [32]. Jung-Hwan et al. in their review discussed the applications of soft-actuated robots based on dielectric elastomer actuators (DEA). In this category of actuators, the authors identified a couple of challenges that have limit their development, such as increased voltage levels for actuating the actuators (which is undesirable for wearable applications), the increased amplitude of motion, and power output [33]. 

Another soft robot with crawling locomotion was designed by Mc Caffrey et al. and is driven by shape memory alloys (SMAs) [34]. Li et al. developed an eight-spring-driven circular robot with SMAs and flexible sensors with closed-loop control [35]. Another case is represented by a pipeline exploration robot based on a crawling locomotion soft robot, which is actuated by three fluidic actuators with open-loop control; this was designed by Zhang et al. [36]. Zhou et al. proposed a gripper based on fluid actuators that have granules in the structure to provide passive variable stiffness during body–finger contact [37]. Calderón et al. proposed a type of robot inspired by earthworm locomotion that is based on two radial and one axial pneumatic actuator and an artificial skin sensor. The control is based on an Arduino Mega microcontroller on which the control strategy of the pneumatic components and sensors of the robot is based [38]. Gu et al. proposed a fluid actuator whose chambers are inclined at a given angle across the actuator surface and, based on this configuration, the actuator was capable of combined bending and twisting motions [39]. Instead, Hu et al. developed two actuator configurations, one with tilted cameras 3D-printed on the whole actuator surface and one with a hybrid actuator with tilted and non-tilted cameras that can be configured according to the specific application [40]. Jizhuang et al. developed a soft robot based on frog locomotion that is driven by fluid actuators, and the robot is capable of linear displacements and rotations [41]. Tang et al. were inspired by the kinematics of cheetahs’ spines during galloping and created a bio-inspired robot based on this principle. The robot is driven by fluid actuators that are connected through hoses to an air supply and has an open-loop control system [42]. Coral W et al. developed a fish-like robot driven using shape memory alloys (SMA) that is equipped with bending and current sensors to help control the robot [43]. Berg et al. made an open-source cable-driven fish from a DC motor with a gear mechanism [44]. 

Shintake et al. developed a fish-like robot with dielectric elastomer actuators [45]. Deng et al. developed a robotic table that can manipulate various objects in the xoy plane by deforming the contact surface. The deformable table is composed of 25 individual pneumatically actuated modules controlled via solenoid valves and an Arduino microcontroller [46]. Chen et al. developed a cube-shaped soft robot that performs locomotion by rolling where the driving is based on an inertial measurement unit (IMU) that identifies the surface that is in contact with the ground; the actuation is performed by fluid actuators [47]. The locomotion of quadrupeds inspired Li et al. to make an autonomous four-legged robot that is not connected to an external power source, thus giving it an increased workspace. The legs are based on a hybrid drive composed of fluidic actuators and nylon cable-based actuators, as well as servo motors [48]. Referring to the manufacturing technologies used in the field of soft robotics, Schmitt et al. discussed the state of the art in the field of soft robot manufacturing methods. From the diverse applications they reviewed, the manufacturing methods most often identified were molding manufacturing methods involving injection molds and additive manufacturing (also called 3D printing) [49]. Additive manufacturing technology applied in the manufacture of soft robots was reviewed in detail by Stano et al., who found three approaches to the use of additive manufacturing in the field of soft robots. These three approaches are related to the realization of injection molds by 3D printing processes, hybrid 3D manufacturing, and full additive 3D manufacturing (modular and monolithic). They also found that the use of 3D printing needs to move from a passive approach involving only the making of molds or other related components to a hybrid or fully additive approach in which soft robotic structures are entirely made by the 3D manufacturing process [50]. Gul et al. in their review analyzed the main challenges of using 3D printing technologies to make soft robots. These challenges are related to the fabrication of fully 3D printed soft robots, limited soft materials, challenges related to printing with multiple materials, and issues related to adhesion between materials [51]. Hann et al. discussed 4D printing in soft robots in their review. They identified certain approaches related to the choice of shape memory material (SMM), more specifically shape memory polymers (SMP), and the diversification of the range of materials with shape memory properties for as many reversible actuations as possible [52].

#### 3.2.2. Aspects Concerning the Open-Loop and Closed-Loop Control of Soft Robots

**Figure 4:**
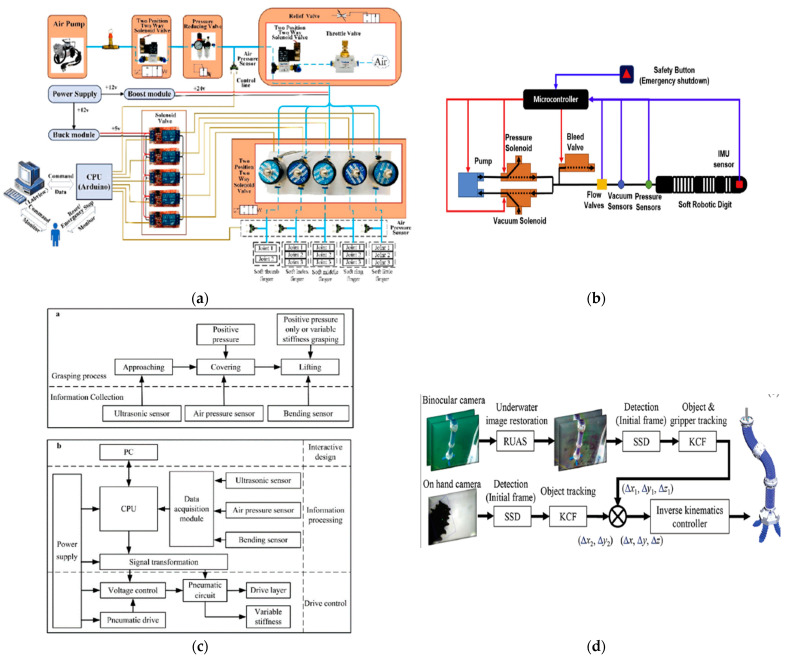
(**a**) Control using vacuum actuation; reproduced with permission [28]; published by ELSEVIER, 2019.; (**b**) diagram of a fluid actuator hand control scheme; reproduced with permission from [53]; published by ELSEVIER, 2020; (**c**) prehensor control based on a fluid actuator with scales inspired by pangolin skin structure; reproduced with permission from [54]; published by ELSEVIER, 2020; (**d**) control scheme of a manipulator with joints based on rotating fluidic actuators [55].

In the paper by Liu et al., the robot driving system was based on closed-loop robot driving. Data from the EGaIn sensor mounted on the robot is collected by the Arduino UNO development board, which drives a servo motor via a PWM signal, driving the 1 mL syringes that supply air to the robot for locomotion [22]. Zhang et al. used both control variants (closed-loop, open-loop). A closed-loop was used for adjusting the water drive system of the propulsion system, as well as the orientation actuators, and robot control was performed in an open loop as there was an IMU sensor mounted on the manipulator end used for its calibration [27]. Feng et al. also approached the control of robotic hands through two control components: one with precise control of pressure and flow that pressurizes the fingers and one with control based on the human–computer interface (realized in Labview software). An Arduino UNO development board was used as the information processing unit to control the process of manipulating objects for people with upper limb disabilities, as shown in Figure 4a [28]. Jaryani et al. approached a similar method of control but, due to the specificity of the application, they also used vacuum actuation to meet the rehabilitation needs of the patients (Figure 4b) [53]. Sun et al. approached the control of autonomous prehension from the perspective of three levels of control: actuation, information processing, and user interface. The use of sensors makes the prehensor possess some level of autonomy, but the prehensor control is limited due to comparison with the existing database that validates the action depending on the object visible (Figure 4c) [54]. Gong Z. et al.’s approach to the manipulator and prehensor kinematic control method for collection activities in aquatic environments was based on inverse kinematics with closed-loop control for two-dimensional and three-dimensional trajectory tracking using video cameras, as shown in Figure 4d [55]. A similar approach with a dynamic manipulator control was proposed by Thuruthel et al. [56]. Xing Z. et al. proposed a manipulator with five modules made of PET and flexible plastic driven by dielectric elastomers. The control is an open-loop type of control that is effectuated by a custom controller consisting mostly of a PLC and high-voltage relays [57]. Yang et al. developed a pneumatically actuated manipulator through pressurization and the use of a vacuum that used joints based on rotary actuators; the manipulator employed closed-loop control with a positioning accuracy of less than 1 cm [58]. 

Nguyen et al. developed a pneumatically operated manipulator with a built-in gripper for handling tasks with various objects. The manipulator is positioned on the person’s body, representing an upper third limb. It is controlled by the user via a joystick and is equipped with EMG sensors to capture muscle intention [59]. Cheng et al. proposed a manipulator based on SMA actuators that has nine degrees of freedom and closed-loop control that employs gyroscope and accelerometer modules [60] or manipulators driven by SMA coils and Hall sensors [61]. Li et al. proposed an SMA-driven manipulator position control method based on fuzzy delay algorithms to increase manipulator accuracy due to the nonlinear hysteretic behavior of SMAs [62]. Jizhuang et al. approached the control of the frog robot through an open-loop control system that connected an HC-12 module to the robot microcontroller, which allowed the robot to be controlled from a PC. The drive system is specific to pneumatic actuators and the robot has high autonomy while not being tied to an external power source [41].

#### 3.2.3. Soft Robots with Applications in Medicine

Highly compliant materials in the structure of soft robots offer great potential for the development of medical equipment and devices due to their mechanical simplicity and a high degree of similarity to the structures and tissue of living organisms. Jen-Hsuan et al. in their review discussed recent achievements in the field of soft robot applications in the medical field. For minimally invasive surgery applications, soft robotics accelerated the development in this field through intrinsic properties, and for rehabilitation and assistive devices, soft robotics greatly improved biocompatibility. In the medical field, soft robotics offers another approach based on safety and efficiency in human–device interaction [63]. Yarali et al. in their review discussed the potential of soft robots made of magneto/electro-responsive polymers (MERPs) in medical engineering, such as their use in drug delivery applications in the human body or artificial tissues. The use of MERPs in biomedical engineering has great potential for development, but to determine the behavior of MERPs in in-vitro environments additional studies are needed [64]. Additionally, Eshaghi et al. confirmed in their review of soft magnetic robot applications that these are still in their infancy and offer great potential in biomedical and non-biomedical applications; however, further studies in both in-vivo and in-vitro environments are needed [65]. According to Hyegyo et al., in the field of hybrid soft robots with nanomaterial, 2DLMs (two-dimensional layered materials) or liquid crystals that have responsive behavior to external stimuli are limited in terms of their integration into real applications. The most advanced soft robots in this field are “stuck” in a conceptual state due to nonlinearity, response time, and prediction of shape deformation under certain stimuli, these being just some of the challenges faced by this field [66]. Another material that is being used more and more due to its properties, and which is still in its infancy, is hydrogel-based soft robots. This material has high elasticity, transparency, ionic conductivity, and biocompatibility; however, these soft robots need new approaches if they are to be integrated into real applications [67]. A new series of liquid metal (gallium)-based soft robots has been developed that possesses flexible sensors and actuators for biomedical and non-medical applications. These materials are increasingly used due to their good electroconductivity and high elasticity [68]. Graphene is also another material with promising characteristics for soft robotics, especially in making sensors and actuators with improved sensitivity and selectivity. Limitations in this field are related to the high-quality production of graphene, compatibility with other materials, and the use of graphene-based soft robots in industrial environments [69]. Textiles integrated into soft robotics have had a significant increase in application and improved technical characteristics; however, the efficiency and characteristics of soft robots with textile structures in practical applications are limiting [70].

**Figure 5:**
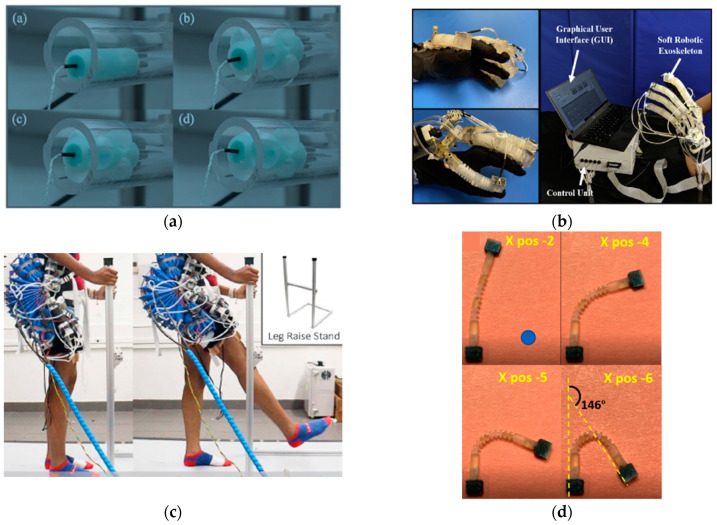

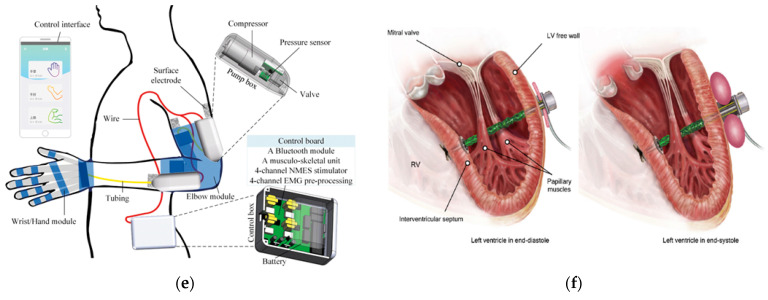
(**a**) Medical robot designed to treat ear diseases [71]; (**b**) rehabilitation glove; reproduced with permission from [53]; published by ELSEVIER, 2020; (**c**) robotic device for hip joint rehabilitation using rotating fluid actuators [76]; (**d**) multimodal locomotion robot for drug delivery; reproduced with permission from [77]; published by ELSEVIER, 2020.; (**e**) wearable device for upper limb recovery after stroke [78]; (**f**) ventricular assist device with McKibben actuator [81].

Lindenroth et al. proposed a medical robot for treating ear diseases that is designed to identify and inject medication precisely without unwanted movements that cause pain to the patient. This is achieved by locomotion within the ear canal utilizing six fluidic actuators that, through combined actuations, perform positioning and orientation movements. So as to detect the optimal injection area, a detection system was developed using a miniature camera, as shown in Figure 5a [71]. Jaryani et al. developed a glove-like exoskeleton for hand rehabilitation using fluid actuators with semi-rigid segments resembling the structure of human fingers. Each finger is actuated by individual pressurization and vacuum through proportional solenoid valves. In addition to pressure and the vacuum sensors, IMU sensors mounted on the fingertips were used to provide feedback to the control system (Figure 5b) [53]. Heung et al. proposed a wearable hand rehabilitation glove for people with stroke. The glove consists of five pneumatically actuated fiber-reinforced fingers. Its control is based on solenoid valves that pressurize or depressurize fluid actuators [72]. Bützer et al. and Burns et al. also developed an exoskeleton for hand rehabilitation that is operated by cables only, which is intended for people who have suffered a stroke or spinal cord injury (SCI) [73,74]. In colorectal cancer, McCandless et al. proposed a soft robotic sleeve to increase navigation safety during the colonoscopy process. The robot attaches to the endoscopic device and provides feedback via optical sensors. Additionally, at a certain value set by the physician via the GUI (Graphical User Interface) in Matlab, the robot will pressurize the three circularly arranged actuators to redistribute pressure over a larger area during navigation [75].

Hip flexion rehabilitation was investigated by Miller et al., who proposed a robotic device based on rotating fluid actuators that is controlled by myoelectric signal capture and IMU sensors (Figure 5c) [76]. In the paper by Joyee et al., a soft robot with multimodal caterpillar-like locomotion is realized, which operates unconnected to an external power source. The robot was 3D printed by a special magnetic field stereolithography process (M-PSL) and was designed to deliver drugs into living organisms, as shown in Figure 5 (d) [77]. Controlled using EMG signal capture, Nam et al. developed a device composed of two elements designed for elbow and hand joint rehabilitation (Figure 5e) [78]. Lindenroth et al. proposed a robot for ultrasound medical imaging based on fluid actuators that provide safe interaction between the device and the patient. Position control is performed in a closed loop based on an electromagnetic tracking sensor and a six-axis NANO 17 force/torque sensor, all guided by a joystick by the physician [79]. Thai et al. proposed a flexible soft robot with applications in surgical medicine. It has a simple configuration as it is driven based on a soft microtube artificial muscle (SMAM) actuator composed of a flexible silicon microtube and a coil [80]. Saeed et al. proposed an implantable ventricular assist robot to increase left ventricular contractions. It uses a McKibben artificial muscle-type pneumatic actuator, as shown in Figure 5f [81]. Considering esophageal cancer, Bhattacharya et al. proposed an endoprosthetic stent-like soft rehabilitation robot for people suffering from dysphagia due to the mentioned disease. The stent is based on a 12-layer fluid actuator, with each layer having four chambers arranged circularly. When pressurized, the chambers expand and block the cross-section of the food passage. The control system is based on the use of 12 proportional valves that pressurize each layer of the stent [82]. Dang et al. developed a biological-like gastric simulator based on simulated gastric peristaltic contractions and the principles of soft robotics. The contractions are performed by pneumatic actuators and the manometry process was used to monitor contractile force [83].

## 4. Conclusions and Future Directions

In this paper, the field of soft robotics has been analyzed from both quantitative and qualitative perspectives. The quantitative analysis was based on a bibliometric analysis of the field of soft robotics concerning its evolution in the 2008–2022 period. Four databases (WOS, ScienceDirect, IEEEXplore, SpringerLink) and a specialized journal titled “*Soft Robotics*” were searched, resulting in a total number of 7646 articles. From the graph analyzing the evolution of the field (Figure 1), the number of articles has increased considerably since 2018. This is based on the intensification of research in the field due to the rapid evolution of related fields, such as 3D printing and materials engineering. Additionally, this increase is also the result of the identification of new applications for soft robots. We believe that future trends will continue until the field reaches full maturity and then saturation. The bibliometric analysis was carried out on the WOS database, specifically the Core Collection. Only research and review articles were included in the analysis of the 2008–July 2022 period, thus the number of publications included in the analysis was 3681. In this analysis, numerous characteristics related to the WOS domains that contributed most to the field, namely authors, countries, productive journals, and most cited articles on WOS, were analyzed in terms of the number of publications. The analysis shows that the field of “Materials Science Multidisciplinary” contributed the most publications, followed by the field of “Robotics”. The most productive journal was “*Soft Robotics*” with more than 450 articles. In terms of countries and productive authors in the field, China and the USA were at the top with a close number of articles, and their productive authors also contributed more than 1% of the total number of publications. The article by Rus et al. [3] had the highest number of citations with more than 2500 citations on WOS. 

The qualitative analysis was the second component addressed in this paper and was based on a total of 111 research and review articles in the 2018–July 2022 timeframe. The articles were identified from four international databases and a peer-reviewed journal based on the search phrase “Soft Robotics”, which resulted in a total of 6400 articles. Due to the large number of articles identified, the selection of articles was conducted in two stages to increase the relevance of this study. The selection of articles was based on a set of clear criteria for inclusion in each selection stage. Table A1 (Appendix A) provides a general analysis of the bibliographic references, specifying the field of application, the materials, the manufacturing technologies, and the main elements in the structure (actuators, sensors). Analysis of the 111 articles was treated from the perspective of three areas of interest: design (biologically inspired soft robots), functionality (open-loop and closed-loop control of soft robots), and applications (soft robots with applications in medicine). The 111 selected bibliographical references have also been analyzed in tabular form according to the materials (Table A2), actuators (Table A3), manufacturing technologies (Table A4), modeling methods (Table A5), and sensors (Table A5) used (Appendix A). As a result of the analysis, some conclusions have been identified regarding the main issues specific to soft robots, and the limitations of each technology and future directions in this area are highlighted below.

It is a certainty that the field of soft robotics is in continuous development given the number of publications and previous reviews, including the present one. According to the present review, the field of soft actuators has developed considerably, especially their operation and properties, and there is a wide range of actuation methods. The most common actuators encountered in the analysis were fluidic actuators of various types, configurations, and reinforcements, which were most often actuated by pressurization and less often by vacuum (or both simultaneously). Use of a specific type of actuator was determined by the specific application. Other common actuation methods included electrically actuated actuators, such as dielectric elastomers (DEA), and shape memory alloy (SMA)-based actuators. Each of these actuation methods has advantages and disadvantages and the choice of an actuator variant requires identification of the optimal characteristics concerning the specific application. The problems found in the analysis are still related to limited force output and limited lifetime.

Concerning the sensors currently used in soft robotics, sensors with a direct role in capturing information from the soft robot by being integrated into the robot’s structure and deforming with the robot structure are predominantly used. These are specifically liquid metal-based sensors (EGaIn) and flexible bending sensors. Regarding sensors with an indirect role (those capturing data from the experimental setup of the robot), pressure, force, current, voltage, laser, ultrasonic, and video camera sensors are most often found. Direct role sensors (the flexible ones) do not offer many options for applications and face various limitations in terms of accuracy, sensing range, and sensor linearity.

Concerning the manufacturing methods of soft robots, the methods most often identified in this review and other similar works are molding methods that use molds and 3D printing. Casting technology offers advantages in terms of part complexity; however, manufacturing time is longer. In the case of 3D printing, future research directions identified in the literature are related to the transition from the 3D printing of molds to full 3D printing of soft robots; however, this requires the realization of new soft materials, simultaneous printing with different materials, and solutions to issues related to their behavior and adhesion. Steps have been made towards full 3D printing with soft materials and 3D printing processes that realize soft structures, such as soft lithography or magnetic field stereolithography (M-PSL), these being some of the new manufacturing technologies identified that may offer new opportunities for the realization of soft robots.

From the perspective of materials used in soft robots, there is a considerable variety available. In the present analysis, most of the materials used were elastomer-based materials, and in this category we identified Ecoflex and DragonSkin bi-component silicone materials from Smooth-On being used in the molding process. Common materials identified in the analysis of 3D printing included acrylonitrile butadiene styrene (ABS) and polylactic acid (PLA), which were used for making the molds and various semi-rigid components of the robotic structure. The analysis identified certain materials that react to various stimuli that have high potential in terms of the manufacture of medical or non-medical equipment and devices, such as drug delivery, surgery, and rehabilitation devices. These materials also have potential for assistive applications as they are similar to the structures and tissues of living organisms. These materials, such as magneto/electro-responsive polymers (MERPs), hybrid robots with 2DLMs (two-dimensional layered materials) or liquid crystals, hydrogel-based robots, liquid metal (gallium)-based robots, and graphene- or textile-based robots, have great potential in the medical and non-medical field but have several limitations, which has led to them being seen as “stuck” in the testing stages. Magneto/electro-responsive polymers have great potential in drug delivery but, to move beyond the test approach and into real-world applications, additional testing and analysis 3in in-vivo and in-vitro environments is required to accurately determine their behavior in the presence of stimuli [64,65]. Additionally, hybrid robots with 2DLMs (two-dimensional layered materials), nanomaterials, or liquid crystals represent another type of materials that respond to stimuli; however, they are limited in their applications due to being locked into limitations related to nonlinearities, response times, and the prediction of shape deformation under certain stimuli [66]. Another category is represented by graphene-based robots, a material that is increasingly used due to its properties. This material is present in the realization of sensors and soft actuators, making a substantial contribution to improvements in their sensitivity and selectivity [69]. 

There are manifold directions in soft robotics that mainly aim to increase the autonomy and integrability of soft robots so as to achieve the performance of biological organisms, thus exhibiting “natural life artificially” [20]. The key components in achieving this goal are related to control (control algorithms and data processing), flexible sensors, and connecting or tethering the robot by cables or hoses to an external power source, which greatly limits its autonomy and behavior. Analyzing the control component of soft robots, the approaches found in the reviewed publications address both closed-loop and open-loop control in similar proportion, while there are also hybrid approaches that combine the two variants. Concerning closed-loop control, the analysis identified different approaches to controlling soft robots precisely and autonomously. One approach was the use of flexible or bending sensors mounted or integrated into the structure that collected data once the structure had deformed, thereby closing the feedback loop. This approach is somewhat limiting because, as more flexible sensors are integrated to determine motion variations, the difficulty of the control component increases significantly. Another closed-loop control approach identified in the analysis was based on a control algorithm that used image processing, which was realized by integrating video cameras that continuously monitored the deformability state of the robot as a function of the objects it interacted with. Additionally, in the case of soft manipulators where control is an important challenge, control approaches are more focused on kinematic control based on quantitative and qualitative kinematic methods and less on approximate behavioral control methods based on dynamic models that also take into account the influence of forces acting on the manipulator during operation.

Due to the non-linear behavior of elastic materials in the soft robot structure, the modeling methods most often used and identified in the analysis are numerical and experimental modeling methods, while analytical methods are less frequently used. The numerical finite element modeling programs most often used in the analysis were Abaqus (Dassault Systèmes) and Ansys, which offer the possibility of simulating and visualizing the results of analysis. There are also other approaches identified depending on the specifics of the applications, for example, in the case of modular reconfigurable robots, there is a need for a 3D simulation and visualization platform of the behavior of the modules that can shorten design time, reduce costs, and verify the effectiveness of algorithms. 

Based on the present analysis, some future research directions have been identified to improve the future characteristics of soft robots so that they may reach characteristics comparable to those of biological beings while also being feasible in industry or commercially available devices. These directions relate to autonomy, integrability, material capabilities to withstand various environmental stresses, controllability, flexible sensors, actuation methods, and manufacturing methods adapted to soft robots. The first area where further research is needed is related to the autonomy of soft robots, which is currently severely limited by the connection to external power supplies as this strongly affects the robot workspace and negatively influences the behavior of the soft robot. With a focus on achieving these characteristics, there are some limitations related to the miniaturization of the components to be integrated, especially in terms of meeting the dimensional criteria corresponding to biological organisms.

Another direction that implicitly also leads to increased autonomy and requires new approaches in research is related to the closed-loop control or feedback control of soft robots. The use of feedback in the control of soft robots is based on the use of flexible sensors within the external structure of the soft robot that transmit data related to the position and deformation of the robot structure. A limiting factor in the use of closed-loop control is closely related to the flexible sensors used, which offer a limited range of available options and also have important limitations. Another limitation that can hamper control is related to the use of a large number of flexible sensors for the satisfaction of control requirements, thus transmitting a multitude of data that makes it difficult to implement the control algorithm.

Another future research direction is related to the development and improvement of 3D additive manufacturing processes that offer the possibility of making soft robots entirely out of more soft materials, as well as the possibility of making soft robots with integrated internal structures such as sensors. One possible way to realize these robots is through 3D printing methods such as soft lithography or magnetic field stereolithography (M-PSL). To achieve the performance of biological beings in terms of autonomy, integrability, adaptability, and efficient locomotion, soft robots still have many aspects that need to be improved or developed in order to achieve these goals, especially if they are to be used in industrial or commercial applications. These limitations and challenges have been identified and addressed above, while this entire paper has aimed to create an overview of the evolution and current state of research in the field of soft robotics while at the same time highlighting research directions in the field.

## Figures and Tables

**Table 1:** Top 10 research areas in WOS contributing to the field of soft robotics.

No.	WOS Categories	Number of Publications	% of 3681
1	Materials Science Multidisciplinary	1335	36.267%
2	Robotics	1080	29.340%
3	Nanoscience Nanotechnology	650	17.658%
4	Physics Applied	543	14.751%
5	Chemistry Multidisciplinary	492	13.366%
6	Chemistry Physical	440	11.953%
7	Physics Condensed Matter	316	8.585%
8	Instruments Instrumentation	301	8.177%
9	Engineering Electrical Electronic	261	7.090%
10	Polymer Science	214	5.814%

**Table 2:** Top 10 journals that have published the most about soft robotics.

No.	Publication Title	Number of Publications	% of 3681	Impact Factor (2021–2022)
1	*Soft Robotics*	457	12.415%	7.784
2	*ACS Applied Materials and Interfaces*	179	4.863%	10.383
3	*IEEE Robotics and Automation Letters*	131	3.559%	4.321
4	*Advanced Materials*	123	3.341%	32.09
5	*Advanced Functional Materials*	110	2.988%	19.92
6	*Frontiers in Robotics and AI*	97	2.635%	4.331
7	*Advanced Materials Technologies*	96	2.608%	8.856
8	*Smart Materials and Structures*	79	2.146%	3.585
9	*Advanced Intelligent Systems*	73	1.983%	7.298
10	*Bioinspiration Biomimetics*	60	1.630%	2.956

**Table 3:** Top 10 countries that have published in the field of soft robotics.

No.	Country	Number of Publications	% of 3681
1	People’s Republic of China	1183	32.138%
2	USA	1160	31.513%
3	South Korea	272	7.389%
4	England	269	7.308%
5	Italy	240	6.520%
6	Japan	213	5.786%
7	Germany	204	5.542%
8	Australia	160	4.347%
9	Switzerland	154	4.184%
10	Singapore	147	3.993%

**Table 4:** Top 10 authors with the highest number of articles in the field of soft robotics.

No.	Author	Country	Number of Publications	% of 3681
1	Majidi	USA	39	1.059%
2	Cianchetti	Italy	38	1.032%
3	Laschi	Italy	38	1.032%
4	Mazzolai	Italy	35	0.951%
5	Liu	People’s Republic of China	34	0.924%
6	Wang	People’s Republic of China	32	0.869%
7	Wood	USA	31	0.842%
8	Wang	USA	29	0.788%
9	Rossiter	England	28	0.761%
10	Dickey	USA	27	0.733%

**Table 5:** Top 10 most cited articles in the field from 2008 to 2022 on WOS.

No.	Author	Title	Country	Journal	Year	Citations (WOS)
1	Rus et al.	Design, fabrication, and control of soft robots	USA	“*Nature*”	2015	2596
2	Amjadi et al.	Stretchable, skin-mountable, and wearable strain sensors and their potential applications: a review	Switzerland	“*Advanced Functional Materials*“	2016	1641
3	Shepherd et al.	Multigait soft robot	USA	“*Proceedings of the National Academy of Sciences of the United States of America*“	2011	1268
4	Kim et al.	Soft robotics: a bioinspired evolution in robotics	USA	“*Trends in Biotechnology*“	2013	1109
5	Ilievski et al.	Soft robotics for chemists	USA	“*Angewandte Chemie-International Edition*“	2011	1096
6	Wang et al.	Skin electronics from the scalable fabrication of an intrinsically stretchable transistor array	USA	“*Nature*”	2018	1033
7	Tee et al.	An electrically and mechanically self-healing composite with pressure- and flexion-sensitive properties for electronic skin applications	Singapore	“*Nature Nanotechnology*“	2012	1032
8	Dickey et al.	Stretchable and soft electronics using liquid metals	USA	“*Advanced Materials*“	2017	792
9	Kim et al.	Printing ferromagnetic domains for untethered fast-transforming soft materials	USA	“*Nature*”	2018	790
10	Mosadegh et al.	Pneumatic networks for soft robotics that actuate rapidly	USA	“*Advanced Functional Materials*“	2014	767

## Data Availability

The data sets used in this study are available on request from the corresponding author.

## Appendix A

**Table A1:** General analysis of bibliographic references.

Author	Ref.	Field of Application	Manufacturing Technologies	The Material Used	Actuator	Sensor
Liu et al.	[22]	Locomotion	Soft lithography, laser processing	SMP, CuNi, Ecoflex 20, Silgard184 with silk threads and particles	Fluidic actuator—air	EGain
Zhang et al.	[27]	Locomotion	Casting	Dragon Skin 10, 30, Ecoflex -30	Fluidic actuator—water	Pressure, flow, IMU
Feng et al.	[28]	Manipulation	Casting	Ecoflex 00–50, Dragon Skin 30	Fluidic actuator—air	Pressure sensor, bending, micro dynamometer, EMG
Lindenroth et al.	[71]	Medical devices	Casting	Ecoflex 00-30, 00-50, Dragon Skin Fx Pro, Smooth-Sil 960	Fluidic actuator—deionized water	Module camera MD-V1001L-91X
Jaryani et al.	[53]	Medical devices, rehabilitation	Casting	Silicone rubber (XIAMETER RTV-4234-T4)	Fluidic actuator—air	Pressure–vacuum sensor, IMU
Zou et al.	[29]	Locomotion	Casting	Dragon Skin 30, Ecoflex 00-30	Fluidic actuator—air	Pressure sensor, dynamometer
Sun et al.	[54]	Prehension	Casting actuator, 3D printing layer with variable stiffness	Dragon Skin 30, rubber, nylon	Fluidic actuator—air	Bending, force, pressure, ultrasonic
Gong et al.	[55]	Manipulation, prehension	Casting	Dragon Skin 10, 30	Fluidic actuator—air	Stereo camera, video camera
McCandless et al.	[75]	Medical devices	Casting	Ecoflex 00-30, VytaflexTM 20	Fluidic actuator—air	Soft optical sensors, pressure
Xing et al.	[57]	Manipulation	Casting, laser cutting	Conductive carbon grease, PET, flexible plastic, VHB_ 4910	Dielectric elastomer	-
Qin et al.	[23]	Locomotion	-	Polyester fabric, thermoplastic polyurethane	Electrostatic actuator, VASA	Laser sensor, digital
Miller et al.	[76]	Medical devices, rehabilitation	Heat sealing, 3D printing	Nylon fabric coated with thermoplastic polyurethane (TPU)	Rotary fluidic actuator—air	Force, IMU, EMG
Zhou et al.	[37]	Prehension	Casting, 3D printing	Dragon Skin 20, 30	Fluidic actuator—air	-
Joyee et al.	[77]	Locomotion	3D printing, stereolithography (M-PSL)	Spot E elastic, magnetic nanoparticles—EMG 1200	Electromagnetic actuator	-
Li et al.	[31]	Locomotion	Casting	Ecoflex 00–30, silicon dioxide nanoparticles, acrylonitrile-butadiene styrene—ABS	Dielectric elastomer—DE	Force sensor
Calderón et al.	[38]	Locomotion	Casting	Ecoflex 00–50, 00-30, butadiene rubber, fiberglass	Fluidic actuator—air	Liquid metal—galinstan
Nam et al.	[78]	Medical devices, rehabilitation	3D printing	PVC, photopolymer	Pneumatic Artificial Muscles (PAM)	Pressure, EMG
Li et al.	[84]	Prehension	-	-	Pneumatic Artificial Muscles (PAM)	-
Gu et al.	[39]	Drive, prehension	Casting	Elastosil M4601, Ecoflex 00–30.	Fluidic actuator—air	-
Guo et al.	[24]	Locomotion	-	Polyimide—dielectric layer, copper layer—electrode, Sylgard 184—insulation layer, dielectric elastomer (VHB 4910), carbon grease (846-80G)	Dielectric elastomer (DEA), flexible electroadhesive (EA)	Video camera, laser sensor
Venkiteswaran et al.	[25]	Locomotion	Casting	Praseodymium powder (PrFeB), silicone Ecoflex 00-10	Magnetic actuator	-
Hu et al.	[40]	Drive, prehension	3D printing	FilaFlex—thermoplastic elastomer	Fluidic actuator—air	Pressure sensor, force
Sui et al.	[30]	Locomotion	Casting	Silicone Ecoflex 00-50, radial magnets	Fluidic actuator—air	Ultrasonic
Jizhuang et al.	[41]	Locomotion	Casting	Silicone Ecoflex 00-50	Fluidic actuator—air (Cuboid)	Motion sensor, pressure
Perez-Guagnelli et al.	[85]	Medical devices	Casting	Silicone Ecoflex 00-30, polyester	Helicoidal fluidic actuator (SoPHIA)	Distance, force
Sonar et al.	[86]	Control	Casting	Polydimethylsiloxane (PDSM), Sylgard 184	Fluidic actuator—air (SPA)	EGaIn
Caffrey et al.	[34]	Locomotion	3D printing	TangoBlack+, VeroWhite+	Shape Memory Actuator (SMA)	-
Yi et al.	[87]	Drive	3D printing	Flexible thermoplastic polyurethane	Rotary fluidic actuator—air	Pressure sensor, torque,
Coral et al.	[43]	Locomotion	3D printing	Polycarbonate, plastic (ABS), lycra fiber, latex, liquid silicone, silicone paint	Shape Memory Actuator (SMA)	Bend sensor, current, temperature
Yang et al.	[58]	Manipulation	3D printing, bonding by heat pressing	Poplin, thermoplastic polyurethane (TPU), acrylonitrile butadiene styrene (ABS)	Rotary fluidic actuator	IMU sensor, pressure
Seref Kemal Talas et al.	[88]	Drive	3D printing	Polyethylene terephthalate, polylactic acid (PLA), polytetrafluoroethylene (PTFE), polyamide 12, latex	Fluidic actuator—air	Force/torque sensor
Cheng et al.	[60]	Manipulation	3D printing	-	Shape Memory Actuator (SMA)	Gyroscope sensor (MPU)
Herianto et al.	[89]	Drive, prehension	3D printing (FDM)	Thermoplastic polyurethane elastomer	Fluidic actuator—air	-
Li et al.	[62]	Drive	Casting	Silicone elastomer (605, 5HA), Ni-Cr resistive fir	Fluidic actuator—air	-
Youxu et al.	[90]	Locomotion	3D printing	Silicone rubber Ecoflex 00-50, Dow Corning 737, polylactic acid (PLA), liquid metal	Fluidic actuator—air	EGaIn
Yang et al.	[61]	Manipulation, locomotion	Casting	Silicone rubber, silicone gel	Shape Memory Actuator (SMA—Flexinol)	Hall sensor
Lindenroth et al.	[71]	Medical devices	Casting	Silicone rubber DragonSkin 10-NV, SmoothSil 945	Fluidic actuator—air	Force/torque sensor, electromagnetic tracking sensor
Zhang et al.	[91]	Locomotion	Casting, 3D printing	Ecoflex 00-50, Kevlar fiber, adhesive (HJ-420)	Fluidic actuator—air	Pressure
Ohta et al.	[92]	Manipulation	3D printing	Silicone, carbon fiber rods, fiberglass, photopolymer, polyurethane sheets	Fluidic actuator—air	Potentiometer
Thai et al.	[80]	Medical devices	3D printing	Flexible silicone microtube, micro-coil	Artificial muscles with soft microtubules (SAM)	-
Liu et al.	[93]	Manipulation	3D printing (SLS)	Silicone rubber, nylon	Fluidic actuator—air	-
Li et al.	[94]	Medical devices	Casting	Elastosil M4601	Actuator with cables	Micro video camera
Roozendaal et al.	[95]	Devices for increasing comfort	Casting	DragonSkin 30, foam (Octaspring)	Fluidic actuator—air	Pressure sensor, phototransistor
Osamu Azami et al.	[96]	Locomotion	Casting	DragonSkin 30	Fluidic actuator—air/water	Pressure sensor, encoder
Saeed et al.	[81]	Medical devices	-	Flexible silicone microtube, fiber	Pneumatic Artificial Muscles (McKibben)	-
Khan et al.	[97]	Drive	Casting	DragonSkin 10	Fluidic actuator—air	Bend sensor, pressure
Li et al.	[32]	Locomotion	Assembly	Acrylic elastomer (3M—VHB), polyethylene terephthalate (PET), electros—carbon grease	Dielectric elastomer (DE)	-
Pengfei Yang et al.	[98]	Locomotion	Casting	Ecoflex 00-30, paper	Fluidic actuator—air (PNs)	-
Digumarti et al.	[99]	Locomotion	Casting	Dragon Skin 10 SLOW, Sil-Poxy, Silk-Pig pigments	Fluidic actuator—air	-
Kang et al.	[100]	Medical devices, rehabilitation	Casting	Polymer (KE-1300T)	Actuator with cables	Sensor PliancyHand Mat (Novel)
Chen et al.	[101]	Drive	Casting	ELASTOSIL RT 622 A, methyl methacrylate, Kevlar fibers, glass	Fluidic actuator—air	Flexible sensor
Wang et al.	[102]	Locomotion	3D printing	Elastomer	Fluidic actuator—air	-
Jiang et al.	[103]	Locomotion	3D printing, (FDM) flexoskeleton	Acrylonitrile butadiene styrene (ABS), polylactic acid (PLA), polycarbonate (PC), adhesive (cyanoacrylate)	Microservomotors	-
Bützer et al.	[73]	Medical devices, rehabilitation	3D printing, sssembly	IP 600 (Igus), stainless steel bands, leaf springs (Precisinox SRL)	Actuator with cables	Bend sensor, force, EMG, EEG
Li et al.	[104]	Control	3D printing	Polymer	Shape Memory Actuator (SMA)	Displacement sensor
Li et al.	[105]	Drive	Casting	Silicone rubber (HC—920), thermoplastic polyurethane (TPU), fibers	Fluidic actuator—air	-
Yang et al.	[106]	Drive	Casting	Ecoflex 00-50, glass particles, paper	Fluidic actuator—air	-
Paternò et al.	[107]	Manipulation	Casting	Ecoflex 00-30, Ecoflex 00-50	Fluidic actuator—air	Pressure sensor
Moghadam et al.	[108]	Locomotion	Laser cutting	Thermoplastic polyurethane (TPU)	Fluidic actuator—air	Pressure sensor
Tang et al.	[42]	Locomotion	Casting, 3D printing	Ecoflex 00-50, polylactic acid (PLA)	Fluidic actuator—air	Pressure sensor
Sayed et al.	[109]	Locomotion	Casting, laser cutting	Dragon Skin 10, 20, 30, Ecoflex 00-10, 30, 50, acrylic polymer	Fluidic actuator—air, electromagnetic induction	Pressure sensor, temperature, omnidirectional sound, distance
Wang et al.	[110]	Locomotion	Assembly	Latex, cotton fiber, polyester fiber	Artificial pneumatic muscles (Curl—CPAM)	Pressure sensor
Niu et al.	[26]	Locomotion	Casting	Ecoflex 00-50	Magnetic actuator	-
Nguyen et al.	[111]	Sensory	Casting	Dragon-Skin 00-30, nylon fibers	Fluidic actuator—air	Pressure sensor, laser, soft sensor
Deng et al.	[46]	Locomotion, manipulation	3D printing	Ecoflex 00-30	Fluidic actuator—air	Pressure sensor
Thuruthel et al.	[56]	Manipulation	-	Elastomer	Fluidic actuator—air	-
Singh et al.	[112]	Manipulation	Casting	Polyamide (PA12)	Fluidic actuator—air	Optical sensor, video camera, potentiometers
Eder et al.	[113]	Manipulation	Assembly	Elastomer	Pneumatic Artificial Muscles (PAM)	Pressure sensor, stretch, gyroscope, 6D accelerometer
Burns et al.	[74]	Medical devices, rehabilitation	Assembly	Textiles (Glove)	Actuator with cables	EMG, flexible sensor
Li et al.	[35]	Locomotion	Assembly	Thin steel, elastomer	Shape Memory Actuator (SMA)	Flexible sensor
Berg et al.	[44]	Locomotion	Casting, assembly, 3D printing	Polylactic acid (PLA), silicon, nylon, PETG, nitrile rubber, POM	Actuator with cables	-
Coad et al.	[114]	Exploration	3D printing	Polythene (LDPE)	Fluidic actuator—air	-
Heung et al.	[72]	Medical devices, rehabilitation	Casting	Dragon Skin 30, start stainless steel	Fluidic actuator—air	-
Bhattachara et al.	[82]	Medical devices	Casting	Ecoflex 00-30	Fluidic actuator—air	Pressure, Force Sensing Potentiometer (FSP)
Chen et al.	[47]	Locomotion	Casting	Ecoflex 00-30	Fluidic actuator—air	IMU
Shintake et al.	[45]	Locomotion	Casting, pad printing	Polymethyl methacrylate (PMMA), polyethylene (PET), Nusil CF19-2186, Sylgard 184, Sylgard RTV-734, carbon black	Elastomer dielectric	-
Liu et al.	[115]	Prehension	3D printing, assembly	ABS, latex, leaf spring	Fluidic actuator—air—hybrid (FHPA)	Force sensor—6D, gyroscope.
Kim et al.	[116]	Manipulation	3D printing, casting	Dragon-Skin 10, polymer	Fluidic actuator—air	EGaIn
Nguyen et al.	[59]	Manipulation	3D printing, casting	ABS, Dragon-Skin 30, Sil-Poxy	Fluidic actuator—air	Pressure sensor, IMU, EMG
Hoang et al.	[117]	Prehension	3D printing, casting	Ecoflex 00-30, carbon grease	Fluidic actuator—air	EGaIn, pressure, force
Dang et al.	[83]	Medical devices	3D printing, casting	Ecoflex 00-30, Sil-Poxy, cyanoacrylate, beeswax	Fluidic actuator—air	Pressure sensor
Ji et al.	[118]	Locomotion	3D printing	Filaments NinjaFlex	Actuator with cables	IMU, TOF (Time of Flight) sensor
Gharavi et al.	[119]	Prehension, rehabilitation.	3D printing, casting	Silicone RTV-2 325, reinforced with fibers	Fluidic actuator—air	Bend sensor
Wu et al.	[120]	Locomotion	3D printing (stereolithography), casting	Ecoflex T606, metal powders (Nd2Fe14B)	Magnetic actuator	-
Wu et al.	[121]	Locomotion	Casting	Silicone rubber, polyacrylate	Fluidic actuator—air	Magnetometer sensor (3 axes)
O’Neill et al.	[122]	Medical devices	Assembly	Textile, polyurethane	Fluidic actuator—air	Torque sensor, pressure
Li et al.	[48]	Locomotion	3D printing, casting	Silicone rubber, thermoplastic urethane (TPU), nylon fibers, fibers	Fluidic actuator—air, actuator with cables	Force sensor, pressure
Zhang et al.	[36]	Locomotion	Casting, 3D printing	Ecoflex 00-50, Ecoflex 00-30, nylon fibers	Fluidic actuator—air	Pressure sensor, force, electromagnetic tracking sensor (EM—6 DOF)
Horvath et al.	[123]	Medical devices	Casting, 3D printing	Medical mesh (Parietex), lycra, velcro, Dragon-Skin FX-Pro	Shape Memory Actuator (SMA)	Pressure sensor
Liu et al.	[124]	Prehension	3D printing	Thermoplastic elastomer (BootFeeder)	DC motor (Sumotor 37GARH)	Force sensor
Cao et al.	[125]	Locomotion	Assembly, casting	Membrane VHB 4910, carbon grease, polyethylene terephthalate (PET)	Dielectric elastomer (DEA), electroadhesion	-
Hofer et al.	[126]	Manipulation	Assembly, 3D printing, laser cutting	Fabric poplin, thermoplastic polyurethane (TPU), thermoplastic adhesive, velcro	Fluidic actuator—air	Pressure sensor

**Table A2:** Analysis of bibliographic references according to the materials.

Ref.	Material	Features	Functionality	Application
[22]	SMP, CuNi, Ecoflex 20, Silgard184 with silk threads and particles	High pressures and forces	Actuator, sensor	Locomotion
[27]	Dragon Skin 10, 30, Ecoflex -30	Fiber-reinforced	Actuator	Biomimetic
[28]	Ecoflex 00–50, Dragon skin 30	Fiber-reinforced—Kevlar	Actuator	Manipulation
[53]	Silicone rubber (XIAMETER RTV-4234-T4)	-	Actuator	Rehabilitation
[29]	Dragon Skin 30, Ecoflex 00-30	Materials with different elasticity to provide stability to the modules	Casing, actuator	Locomotion
[54]	Dragon Skin 30, rubber, nylon	Reinforced with glass fiber	Actuator	Prehension
[55]	Dragon Skin 10, 30	Fiber-reinforced, Shore A hardness of 10, 30	Actuator	Manipulation, prehension
[75]	Ecoflex 00-30	Low RI (refractive index)	Actuator, main body	Medical devices
[57]	Conductive carbon grease, PET, flexible plastic, VHB_ 4910	Constructive simplicity	Structure, actuator	Grasping devices, manipulation
[38]	Ecoflex 00–50, 00-30, butadiene rubber, fiberglass	Actuators reinforced with glass fibers	Actuator	Locomotion
[39]	Elastosil M4601, Ecoflex 00–30	-	Actuator	Drive, prehension
[25]	Silicon Ecoflex 00-10	Low elastic modulus, high elongation at break	Mixing polymer material	Locomotion
[40]	FilaFlex—thermoplastic elastomer	High elasticity, abrasion resistance, low modulus of elasticity	Robot body	Drive, prehension
[43]	Lycra fibers, latex, liquid silicone, silicone paint	It gives the robot fish mobility, waterproofing, and toughness	Outer layer	Locomotion
[58]	Poplin, thermoplastic polyurethane (TPU)	High tensile and tensile strength	Structure of actuators	Manipulation
[88]	Polyamide 12	High tensile strength, low density	End effector	Drive
[32]	Acrylic elastomer (3M—VHB), polyethylene terephthalate (PET), electros—carbon grease	Good compliance, flexibility, manufacturing, and actuation	Robot body	Locomotion
[98]	Ecoflex 00-30	-	Actuator	Locomotion
[100]	Polymer (KE-1300T)	Low deformability	Actuator body	Medical devices
[108]	Thermoplastic polyurethane (TPU)	Inexpensive commercially available materials	Robot body	Locomotion
[42]	Ecoflex 00-50, polylactic acid (PLA)	-	Robot body	Locomotion.
[45]	Polymethyl methacrylate (PMMA), polyethylene (PET), Nusil CF19-2186, Sylgard 184, Sylgard RTV-734, carbon black	-	Robot body, actuator	Locomotion
[59]	ABS, Dragon-Skin 30, Sil-Poxy	Reinforced with plastic rings	Robot body	Manipulation
[83]	Ecoflex 00-30, Sil-Poxy, cyanoacrylate, beeswax	Composed of 4 segments	Body simulator	Medical devices
[48]	Silicone rubber, thermoplastic urethane (TPU),nylon fibers, fibers	Fiber-reinforced	Leg structure	Locomotion
[126]	Fabric poplin, thermoplastic polyurethane (TPU), thermoplastic adhesive, velcro	Bonding the layers with a heat press	Actuator	Manipulation

**Table A3:** Analysis of bibliographic references according to the actuators.

Ref.	Actuator Type	Mode of Driving	Driving System	Power Density	Weight	Application	Limitations/Challenges	Characteristics
[22]	Fluidic actuator—air	Pressurization	Arduino Uno, EGaIn sensor, servo motor	-	0.45 g	Locomotion	Influence of wires and connecting tubes on locomotion	Structure made up of 5 layers with thicknesses of 20 μm
[27]	Fluidic actuator—water	Pressurization	Pump, pressure sensor, flow IMU, stepper motor	-	432 g	Biomimetics	-	Semi-round siphons
[28]	Fluidic actuator—air	Pressurization	Pump, pressure sensor, Arduino UNO, EMG	-	-	Manipulation	-	Designed based on human fingers
[71]	Fluidic actuator—deionized water	Pressurization	3 mL syringes, stepper motors, TMCM-6214 controller	-	-	Medical devices	-	Performing rotational and translational movements
[53]	Fluidic actuator—air	Pressurization, vacuumed	Pump, solenoid valves, proportional valves, microcontroller, pressure/vacuum sensor, IMU	-	-	Medical devices, rehabilitation	-	Actuator with semi-rigid segments
[29]	Fluidic actuator	Pressurization, vacuumed	Pump, solenoid valve, actuator, pressure sensor	-	~50-90 g / module	Locomotion	Limited applications due to connecting tubes, no feedback loop	Caterpillar-like locomotion and reconfigurable structure
[54]	Fluidic actuator—air	Pressurization, vacuumed	Air pump, vacuum, Arduino UNO, bending sensor, force, ultrasonic, pressure; proportional valve	-	-	Prehension	Clamping of parts with limited dimensions	Reinforced with glass fiber
[55]	Fluidic actuator—air	Pressurization	Closed-loop control based on stereo camera and video camera	-	1050 g	Manipulation, prehension	Closed-loop control due to the non-linear characteristics of the material	Three degrees of freedom, reinforced with Kevlar fibers
[75]	Fluidic actuator—air	Pressurization	Control using graphical user interface (GUI) and feedback from optical sensors in the form of force	-	-	Medical devices	Reduction in thickness and outer diameter	Three actuators positioned circularly with an angle of 120º
[57]	Elastomer dielectric—electric	Electric	Programmable automatic—(PLC), relay, EMCO amplifier	-	18 g	Manipulation	Reduced handling force	Simple and cheap construction
[23]	Electrostatic servo motor, VASA	Vacuumed, electric	Vacuum regulator, digital sensor, laser	-	43 g	Locomotion	Limited autonomy due to connection to external energy sources	Versatile, fast, and efficient locomotion
[76]	Rotary fluidic actuator—air	Pressurization	Regulator, electropneumatic valve, pump	-	-	Medical devices, rehabilitation	Placing the device on the patient’s torso	Rehabilitation of hip flexion
[37]	Fluidic actuator—air	Pressurization	-	-	460 g	Prehension	Grasping of sharp elements	Variable stiffness using passive particle locking
[77]	Electromagnetic actuator	Electromagnetic	Magnetic foot control, tilt angle measurement with Matlab	-	0.23 g	Locomotion	Attachment of the robot leg mechanism to the substrate	Average locomotion speed of 3.1 mm/s
[31]	Elastomer dielectric—DE	Electric	Power supply, signal generator, voltage amplifier	9 mW/g	4.9 g	Locomotion	Fulfilling the characteristics of autonomy	Constructive simplicity
[78]	Pneumatic muscles	Pressurization	Compressor, valve, pressure sensor, Bluetooth module, EMG, MCU (PIC18F46K22)	-	208 g	Medical devices, rehabilitation	-	Based on the control of EMG signals
[84]	Pneumatic Artificial Muscles (PAM)	Pressurization	Controller, air pump, battery, solenoid valve	-	1.5 kg	Prehension	Low grip speed	Clamping autonomy of 300 cycles
[39]	Fluidic actuator—air	Pressurization	-	-	-	Drive, prehension	-	Making bending and twisting movements by varying the camera angle
[24]	Dielectric Elastomer (DEA), flexible electroadhesive (EA)	Electric	High voltage amplifier, power supply, MOSFET, Arduino UNO	-	12 g	Locomotion	High voltage levels	Crawling on vertical surfaces at a speed of 2.3 mm/s at a frequency of 0.8 Hz
[25]	Magneticactuator	Magnetic fields	6 electromagnetic coils, video camera	-	-	Locomotion	Investigating locomotion in a straight line only	Lack of radiation and not connecting the robot with cables or wires
[41]	Fluidic actuator—air (cuboid, arched)	Pressurization	Arduino Mega 2560, HC-12 module, air pump, battery, solenoid valve, pressure regulator, CO2 tank, motion sensor, pressure	-	1.29 kg	Locomotion	Reducing the overall size and weight of the robot torso	Reinforced with Kevlar fibers
[85]	Helicoidal fluidic actuator (SoPHIA)	Pressurization	-	-	95 g	Medical devices	Implementation of soft sensors to take information from the robot	Wrapped in polyester fabric
[34]	Shape Memory Actuator (SMA)	Electromagnetic.	Signal generator, linear amplifier, coils	-	7 g	Locomotion	Impedance variation limited to the power amplifier	Strong magnetic field for activating SMA wires
[87]	Rotary fluidic actuator—air	Pressurization	Pump, proportional valve, pressure sensor, rotary encoder	-	300 g	Actuators	-	Payload of 18.5 N·m at 180 kPa pressure
[43]	Shape Memory Actuator (SMA)	Electric	Flexible sensor, current, analog/digital converter, SMA fire, microcontroller	-	-	Locomotion	Protection of the robot at the temperature of the SMA	The SMA temperature can reach up to 90 °C
[58]	Rotary fluidicactuator—air	Pressurization, vacuumed	Air pump, vacuum, Arduino Mega 2560 R3, proportional valves, pressure sensor, IMU	-	300 g	Manipulation	Bonding the component layers of the actuator	Rotational articulation of the manipulator
[60]	Shape Memory Actuator (SMA)	Electric	MOS amplifier, gyroscope sensor, linear encoder, microchip STM32 controller	-	-	Manipulation	Additional cooling methods to shorten SMA recovery time	Nine degrees of freedom, good positioning
[61]	Shape Memory Actuator (SMA- flexinol)	Electric	SMA coils, amplifier, Hall sensor, Arduino UNO, PC	-	-	Manipulation, locomotion	Austenitic phase transition temperature	Durable, cheap, and accurate manipulator
[80]	Artificial muscles with soft microtubules (SMAM)	Pressurization	Flexible silicone microtube, micro-coil, optical encoder, syringe, micromotor, Matlab/Simulink	-	0.28 g	Medical devices	The non-linear adaptive control algorithm	Elongation by 245%
[94]	Wired actuator	Electric	Wires, stepper motor, micro-camera, Arduino microcontroller, electromagnetic tracking system, user interface (GUI)	-	-	Medical devices	Controllability of the robot	1.4 ± 0.4 mm positioning and 1.5 ± 1.1 degree orientation accuracy
[95]	Fluidic actuator—air	Pressurization	Pump, pressure sensor, solenoid valve, Arduino	-	-	Devices to increase comfort	The distribution of the surface covered by the device is insufficient	Pressure distribution to ensure people’s comfort
[32]	Elastomerdielectric	Electric	Power supply, high voltage amplifier, relay, microcontroller, video camera	-	12.2 g	Locomotion	Rolling speed, smooth locomotion	Relatively high speed of the robot—0.65 m/s
[100]	Actuator withcables	Electric	Actuator (IG-32GM 03TYPE), processor (TMS320F2808), Li-ion battery, pliancy sensorhand mat (Roman)	-	104 g	Medical devices	Finger joint stiffness	The cables are connected to the glove by tension springs
[73]	Actuator withcables	Electric	DC motor (Maxon), motor drivers (ESCON), Arduino Yun Mini, bend sensor, EMG, battery	-	148 g	Medical devices, rehabilitation	Grasping objects where more dexterity is required	Wearable exoskeleton for daily activities, easy for users to accept
[105]	Fluidic actuator—air	Pressurization	Electropneumatic regulator	-	-	Drive	Large range of motion angle	Air pressures up to 400 kPa
[46]	Fluidic actuator—air	Pressurization	Pneumatic pump, solenoid valves, valves, Arduino	-	-	Locomotion, manipulation	The problems regarding quantification of the deformations led to a failure to solve the model in its entirety	Open-loop driving
[56]	Fluid actuator—air	Pressurization	Micro proportional regulator, Vicon tracking system, controller	-	-	Manipulation	Positioning accuracy	12 degrees of freedom (DOF)
[35]	Shape Memory Actuator (SMA)	Electric	Controller (STM32), flexible sensor, voltage amplifier, wireless module, PC	-	100 g	Locomotion	-	Closed-loop control
[72]	Fluidic actuator—air	Pressurization	Pump, solenoid valve	-	207 g	Medical devices, rehabilitation	Reduced degree of autonomy	Fiber-reinforced
[82]	Fluidic actuator—air	Pressurization	Compressor, proportional valves, Raspberry Pi, AD/DA interfaces, pressure sensors, force	-	-	Medical devices	The occurrence of corrosion in stents	The ROSE actuator has 12 layers
[47]	Fluidic actuator—air	Pressurization	GUI, pump, valve, battery, microcontroller	-	830 g	Locomotion	Reduced size and weight	Cube with a side of 10 cm
[45]	Elastomer dielectric	Electric	High voltage converter, microcontroller, C-MOS camera	-	4.4 g	Locomotion	-	Swimming speed of 37.2 mm/s at 5 kV
[59]	Fluidic actuator—air	Pressurization	Solenoid valve, pressure sensors, IMU, EMG, joystick, controller	-	960 g	Manipulation	Connecting the manipulator through cables and hoses	Controlled by joystick
[120]	Magnetic actuator	Magnetic field	Magnet, camcorder	-	-	Manipulation	Designing the robot to perform operations	Controlled by magnetic field
[36]	Fluidic actuator—air	Pressurization, vacuumed	Arduino Mega 1820, pump, solenoid valves, PC, electromagnetic hatching (EM) sensor, pressure sensor, camera	-	14.5 g	Locomotion	Reduced detection and handling capability	Ability to handle a load 10 times its weight
[126]	Fluidic actuator—air	Pressurization	Solenoid valves, pressure sensor, Labjack T7 Pro, pressure regulator	-	126 g	Manipulation	Limited range of motion	Actuator with three internal cavities (bellows) arranged circularly

**Table A4:** Analysis of references according to the specific technologies.

Ref.	Technology	Component in the Structure	Material	Size	Benefits	Disadvantages
[22]	3D—lithography	Actuator	Silgard184 with silk threads and particles	35 mm long, 12 mm wide, 1 mm thick	Parts with complex geometries	-
[27]	Casting	Actuator	Dragon Skin 10	120 mm long, outer diameter 65 mm, hole diameter 16 mm	Complex parts with internal cavities	-
[53]	Casting	Actuator	Silicone rubber (XIAMETER RTV-4234-T4)	-	-	-
[29]	Casting, 3D printing	Actuator—casting, mold—3D Printing	Dragon Skin 30, Ecoflex 00-30	Length 154 mm	Three degrees of freedom (t-t-r), travel speed 18.5 m/h, rotation 1.63°/s	Limited autonomy due to lack of feedback loop control
[54]	Casting, 3D printing	Actuator	Dragon Skin 30, rubber, nylon	-	-	-
[55]	Casting	Actuator	Dragon Skin 10, 30	540 mm long, 48 mm diameter	Precise positioning thanks to feedback control	High-complexity control system
[75]	Casting	Robot body, actuator	Ecoflex 00-30, VytaflexTM 20	118 mm long, 62 mm wide, 3.5 mm thick	-	-
[57]	Casting, assembly	Actuator	Conductive carbon grease, PET, flexible plastic, VHB_ 4910	Length 320 mm, weight 18 g	Constructive simplicity	Relatively high actuation voltages
[77]	3D Printing—stereolithography (M-PSL)	The body of the robot	Spot E elastic, magnetic nanoparticles—EMG 1200	Length 40 mm	Composite print directly from a digital model	-
[40]	3D printing	Actuator	FilaFlex—thermoplastic elastomer	Length 180 mm, width 25 mm	Parts with complex geometries	Surface quality
[85]	Casting	Actuator, the body of the robot	Ecoflex 00-50	Length 18 cm, width 10 cm, height 6 cm	-	Additional cost
[58]	3D printing, bonding by heat pressing	Actuator	Poplin, thermoplastic polyurethane (TPU), acrylonitrile butadiene styrene (ABS)	-	Fast and cheap model making	Life cycle unknown, optimal structure configuration
[91]	Casting	Actuator	Ecoflex 00-50, Kevlar threads, adhesive (HJ-420)	Length 100 mm	Elongation accuracy of 0.51 mm	Lack of flexible sensors
[32]	Assembly	Robot body (actuator)	Acrylic elastomer (3M—VHB), polyethylene terephthalate (PET), electros—carbon grease	Diameter 106 mm	Relatively high locomotion speed	Relatively high voltage levels
[103]	3D printing—(FDM) flexoschelet	The skeleton of the robot	Acrylonitrile butadiene styrene (ABS), polylactic acid (PLA), polycarbonate (PA), adhesive (cyanoacrylate)	Leg length 70 mm	Fatigue resistance of parts is greatly improved	-
[105]	Molding with built-in core	Robot body (actuator)	Silicone rubber (HC—920), thermoplastic polyurethane (TPU), fibers	Length 940 mm, width 35 mm	It allows the realization of actuators with a wide range of motion	-
[108]	Laser cutting	Robot body (actuator)	Thermoplastic polyurethane (TPU)	Thickness 39 µm	Making robots in a relatively short time	Limited material types

**Table A5:** Analysis of references according to the modelling methods.

Ref.	Component in the Structure	Modeling Method	Size	Power	F/M
[27]	CFRD—central water flow regulation channel	Analytical, experimental	Diameter 16 mm	-	-
[28]	Actuator (finger)	Experimental, numerical	-	-	-
[54]	Actuator	Experimental, numerical	-	-	-
[75]	Robot	Experimental, numerical	Length 118 mm, width 62 mm, thickness 3.5 mm	-	-
[37]	Gripper	Analytical, experimental	-	-	-
[77]	Robot structure	Experimental, numerical	Length 40 mm	-	-
[31]	Robot structure	Experimental, numerical	Length 40 mm, width 10 mm	9 mW/g	-
[38]	Sensor	Experimental, numerical	Length 93 mm, diameter 29 mm	-	-
[39]	Actuator	Analytical, numerical, experimental	Length 104 mm, height 14.5 mm, width 15 mm	-	-
[40]	Actuator	Experimental, numerical	Length 180 mm, width 25 mm	-	-
[30]	Robot mode	Experimental, numerical	-	-	-
[85]	Actuator	Analytical, experimental	-	-	-
[60]	Actuator mode	Experimental, numerical	Diameter 80 mm, length 345 mm	-	-
[89]	Actuator	Experimental, numerical	Length 66.2 mm	-	-
[90]	Actuator	Experimental, numerical	-	-	-
[91]	Actuator	Experimental, numerical	Length 100 mm	-	-
[105]	Actuator	Experimental, numerical	Length 940 mm, width 35 mm	-	-

**Table A6:** Analysis of bibliographic references according to the sensors.

Ref.	Sensor Type	Principle	Material	Characteristics	Application
[22]	EGaIn	Resistance variation by changing the geometry of the microchannels with the elongation of the material	Elastomer, eutectic, gallium, indium	Integrate into the elastic structure	Closed-loop control systems
[54]	Bending, force, pressure, ultrasonic	The variation in electrical resistance with the deformation of the structures	-	Integration into the actuator structure	Closed-loop control systems
[75]	Soft optical sensor	Converts the optical signal into force	Vytaflex 20	Core 100 mm long and 1x1 cross-section	Medical applications
[38]	Liquid metal—galinstan	Resistance variation by changing the geometry of the microchannels with the elongation of the material	Elastomer, eutectic, gallium, indium	Channels of square cross-section with 500 micron sides	Closed-loop control systems
[86]	EGaIn	Resistance variation by changing the geometry of the microchannels with the elongation of the material	Elastomer, eutectic, gallium, indium	Channel thickness of40 µm	Closed-loop control systems
[43]	Bending, current, temperature	The variation in electrical resistance with the deformation of the structures	-	Integration into the actuator structure	Medicine, robotics
[90]	EGaIn	Variation in electrical resistance with sensor deformation	Ecoflex 00-50, elastomer, eutectic, gallium, indium	Tactile ability	Closed-loop control systems
[97]	Bending, pressure	The variation in electrical resistance with the deformation of the structures	-	Mounting on the bottom layer of the actuator	Closed-loop control systems
[101]	Bending	The variation in light intensity through the material	Methyl methacrylate (PMMA)	Integrated on the actuator	Closed-loop control systems
[111]	Soft sensor	Variation in sensitivity at the time of variation in the morphology of the structure	Dragon Skin 00-30	Integrated on the actuator	Sensory systems based on rodent whiskers
[74]	Flexible sensor	The variation in electrical resistance with the deformation of the structures	Polyimide	Integrated on the exoskeleton	Measuring the angle of each joint
[35]	Flexible sensor	The variation in electrical resistance with the deformation of the structures	Polyamide	Positioned on the robot structure	Closed-loop robot locomotion
[115]	Force sensor—6D, gyroscope	Variation in electrical resistance	-	Positioned on the robot structure	Force and angular displacement monitoring
[116]	EGaIn	The variation in electrical resistance with the deformation of the structures	Dragon Skin 10, eutectium, gallium, indium	Positioned on the robot structure	Anti-collision detection sensor
[59]	Pressure sensor, IMU, EMG	Variation in electrical resistance	-	Manipulator control	Manipulation
[117]	EGaIn sensor, carbon grease	The variation in electrical resistance with the deformation of the structures	Ecoflex 00-30	Integrated on the fingers of the gripper	Control system
[119]	Bend sensor	The variation in electrical resistance with the deformation of the structures	RTV-2 325	Integrated into the actuator	Angular variation as a function of pressure
